# Intestinal dysbiosis and allogeneic hematopoietic progenitor cell transplantation

**DOI:** 10.1186/s12967-016-1094-3

**Published:** 2016-12-03

**Authors:** Vikram M. Raghunathan, Iris Sheng, Seah H. Lim

**Affiliations:** 1 Division of Hematology and Oncology, Department of Medicine, Rhode Island Hospital, Room 140 APC Building, 593 Eddy Street, Providence, RI 02903 USA; 2 Brown University Warren Alpert School of Medicine, Providence, USA

## Abstract

The intestinal microbiota is a diverse and dynamic ecosystem that is increasingly understood to play a vital role in human health. Hematopoietic stem cell transplant recipients undergo prolonged exposure to antimicrobials, chemotherapeutic agents, and immunosuppressants, resulting in profound shifts in the gut microbiome. A growing body of research has revealed the ways in which these microbiologic shifts shape immune modulation, affecting susceptibility to infections and graft-versus-host disease, the two major post-transplant complications in this population. As transplant medicine becomes increasingly personalized, the potential for microbiome-modulating treatments holds immense potential. Strategies to preserve the intestinal microbiota, including targeted antibiotics, prebiotics and probiotics, and fecal microbiota transplant could mitigate some of the microbiologic shifts in stem cell transplant recipients, and reduce the incidence of peri-transplant morbidity and mortality.

## Background

The intestinal microbiota, the vast community of bacteria and other micro-organisms inhabiting the human gastrointestinal (GI) tract, plays a vital role in human physiology and health. A growing body of research has examined the dynamic and complex ecosystem of the GI tract and its impact on systemic illness. Since 2007, the Human Microbiome Project has sought to collect genomic information on various human microbiomes, with the intention to expand our knowledge of these active biologic systems to enhance our ability to prevent and treat diseases.

The fundamental characteristic of the intestinal microbiome is its diversity. The GI tract plays host to hundreds of microbial species, which interact with each other and with the environment in countless ways. A major survey of ribosomal RNA (rRNA) sequences from stool and intestinal mucosa by Eckburg et al. identified nearly 400 phylotypes, with a predominance of *Bacteroidetes*, *Eubacterium*, and *Firmicutes*, in healthy adult hosts [1]. Importantly, significant inter-subject variability was noted, consistent with the notion that each human possesses a unique microbiological fingerprint. Many factors may influence this personalized ecosystem, including age, diet, geography, and exposure to medications, particularly antibiotics and chemotherapeutic agents. How each individual’s enterotype affects illness and might affect medical treatment will be an important question in the new age of personalized medicine.

Commensal flora in the intestinal tract are already known to have many crucial functions. Endogenous bacteria directly inhibit pathogens by colonizing available niches and consuming nutrients, and in some cases by secreting antimicrobial peptides. The native bacterial milieu promotes development of intestinal mucosa, and modulates immune response through regulation of inflammatory cytokines and gut antibodies. Intestinal flora also produce enzymes required for the digestion of certain starches, oligosaccharides, and sugars, and aid in absorption of dietary minerals.

Allogeneic stem cell transplant (SCT) recipients are a population at heightened risk of many infectious and inflammatory disorders. Their underlying malignancies, prolonged hospitalizations, and extensive exposure to antibiotics and chemotherapeutic agents make them particularly vulnerable to major shifts in the intestinal microbiome. An understanding of these microbiologic shifts may be crucial in creating strategies to prevent and treat disease in this unique population.

## Main text

### Factors affecting microbiota of SCT recipients

In order to understand how changes in the microbiota affect SCT recipients, it is necessary to examine the baseline characteristics of intestinal flora in this population, and the shifts that occur during the transplantation process. Holler et al. used a combination of 16S ribosomal RNA sequencing and urinary indoxyl sulfate measurements to analyze stool samples of patients for changes in bacterial prevalence during transplantation. They found a standard initial distribution of major microbial phyla across hosts before transplant. The most prevalent phylum was *Firmicutes*, followed by *Bacteroidetes*, *Proteobacteriae*, and *Actinobacteriacea* [2]. Major species of *Firmicutes* included *Eubacterium rectale, Clostridium phytofermentans,* and *Lactobacillus lactis.* The difference between the microbiome of non-transplant donors who had recent hospitalization and SCT patients is in the prevalence of *Enterococcus*. After SCT, there was a 21 percent increase in prevalence of *Enterococcus*, with a notable expansion of *E. faecium* and a complementary decrease in *Firmicutes* and other commensal phyla. A similar microbiologic shift was noted by Ubeda et al. [3] who identified dominance of vancomycin-resistant Enterococcus as a predictor of bacteremia in SCT recipients, and by Eriguchi et al. [4] who observed that active gastrointestinal graft-versus-host disease (GVHD) was associated with a prominent shift towards *E. coli.*

Using a murine model, Jenq et al. found a dramatic loss in microbial diversity in the first two weeks after bone marrow transplant. The loss of diversity became even more pronounced in the setting of GVHD. Analysis of bacterial subpopulations demonstrated an expansion of *Lactobacillales* and *Enterobacteriales*, with a prominent loss of *Clostridiales*. Since these changes were irrespective of whether there was GVHD or not, it was concluded that the pre-treatment with radiation and chemotherapy were responsible for the microbiologic shifts observed after SCT [5].

However, the majority of studies documenting changes in microbiota occurred in the presence of antimicrobial exposure for gut decontamination, which was the standard practice in SCT. This practice came under scrutiny when it was found that the shifts in endogenous flora may have adverse effects on post-transplant complications. Beelen et al. demonstrated that although pre-treatment with the combination of ciprofloxacin and metronidazole resulted in a tenfold decrease in anaerobic bacterial culture growth in recipients of transplants from HLA-identical sibling donors, this was accompanied by an increase in *Enterococcus* [6].

The choice of pre-transplant antimicrobials also plays an important role in shaping shifts in the microbiota. Pre-treatment with metronidazole resulted in the most pronounced Enterococcal predominance in the post-transplant period; conversely fluoroquinolone exposure led to a significant decrease in *Proteobacteria* [7].

It is hypothesized that commensal bacteria induce antimicrobial peptides, which help maintain a healthy, diverse microbiome, and intact intestinal mucosa. With prolonged antibiotic exposure, the loss of commensals is accompanied by decreased induction of microbial-associated molecular patterns (MAMPs), specifically Reg3alpha, the main peptide limiting Enterococcal overgrowth [8]. These MAMPS also communicate with the host immune system via pattern recognition receptors (PRRs). Activation of PRRs increases major histocompatibility complex expression and co-stimulatory molecules on the antigen-presenting cells and endothelial cells. This cascade ultimately leads to the increased production of tumor necrosis factor, type I interferons, and interleukin (IL)-1 and IL-6. A decrease in the activation of this pathway due to decreased level of MAMPs renders the host more susceptible to intestinally originated infections. The intestinal microbiota is also important in promoting differentiation of regulatory T cells, generation of IgA-secreting B cells, and formation of secondary lymphoid organs phenotypes [9]. The liver normally maintains tolerance against these harmless antigens and any commensal bacteria that translocate from the gut. However, when immunosuppressants are used, this homeostasis is perturbed. In addition, immunosuppressive agents, including chemotherapy, can lead to a loss of intestinal microvilli, tight junction damage, and a decrease in IgA secretion. Exposure to chemotherapy, even in the absence of antimicrobial use, is associated with a drastic drop in *Faecalibacterium* and increase in *Enterococcus* [10].

### *Clostridium difficile* colitis

Hospitalized patients, and immunocompromised hosts in particular, are vulnerable to a wide range of nosocomial pathogens. Among these, *Clostridium difficile* is the most common. The emergence of the NAP1/027 strain of *C. difficile* over the first decade of the twenty-first century has been associated with more severe infection [11, 12]. Recent data reveal that *C. difficile* has surpassed Methicillin-resistant *Staphylococcus Aureus* (MRSA) to become the most prevalent nosocomial infection in community hospitals in certain regions of the United States [13].

SCT recipients possess many risk factors for *C. difficile* infection (CDI), including prolonged hospitalization, exposure to broad-spectrum antimicrobials in the peri-transplant period, and long-term immunosuppression. Recent literature show CDI rates among SCT recipients are up to nine times higher than in the general inpatient population [11, 12]. A study by Jain et al. found that 12 percent of patients admitted to their institution for SCT were colonized with toxigenic *C. difficile* [14]. Agha et al. reported that 11 percent of SCT recipients developed peri-transplant CDI [15]. Interestingly, the only factor independently associated with development of CDI was a previous history of the same. Patients with matched-related grafts had a lower likelihood of developing CDI in the study population [15]. A review by Alonso et al. found an overall CDI rate of 9.2% among SCT recipients, with higher rates among allogeneic transplant recipients compared to those who underwent autologous transplant (12.5 vs. 6.5%) [11, 12].

Changes in intestinal microbiota are inherently part of the pathogenesis of CDI. The traditionally understood mechanism involves exposure to broad-spectrum antibiotics altering native gut flora, allowing for colonization by toxigenic *C. difficile*. The shifts in the intestinal microbiome of SCT recipients is likely intrinsically tied to their unique vulnerability to CDI. Cytotoxic chemotherapy given prior to transplant causes disruption of endogenous flora, enabling *C. difficile* colonization. A study by Loo et al. demonstrated that prior chemotherapy increased the likelihood of *C. difficile* colonization over twofold [16]. The eradication of endogenous flora by preparative chemotherapeutic regimens opens a larger microbiological space than exposure to standard antimicrobials, leaving a wider available niche in these patients. The discovery that matched-related hematopoietic grafts were associated with a lower risk of CDI than matched-unrelated grafts suggests that pre-existing microbiome characteristics play a role in determining CDI susceptibility [15]. Pairing donors and recipients with similar gut microbiota, and probable similar past environmental exposures, may produce less disruption to endogenous flora after transplant.

### Graft-versus-host disease

Acute GVHD is a leading cause of morbidity and mortality among SCT recipients. The changes in intestinal microbiome associated with SCT are thought to play a role in the development of GVHD. The pathophysiology of GVHD involves host antigen-presenting cells activating donor T-lymphocytes, leading to a hyper-proliferative inflammatory cascade and tissue damage. There has long been speculation about the role of intestinal environment on GVHD; early studies suggested that antimicrobial prophylaxis may reduce the incidence of GVHD, though these findings were not replicable [6]. Holler et al. found that at time of admission for SCT, patients showed a similar distribution of dominant commensal strains. With exposure to pre-transplant antimicrobials, all recipients experienced major changes in intestinal microbiota, with an overall loss of bacterial diversity. There was a notable expansion of *Enterococcus* species with a decreased in other phyla [2]. This shift was most pronounced in patients with active GI GVHD.

There is also evidence that specific commensal organisms may play an outsized role in modulating GI inflammation. In one cohort study, decreases in anaerobic bacteria in fecal samples, and in particular *Blautia* spp., were correlated with higher rates of acute GVHD and higher mortality [17]. Studies of intestinal flora in SCT patients with GVHD have shown that a reduction in microbial diversity was associated with higher mortality, and that loss of intestinal bacterial diversity is an independent predictor of mortality in SCT recipient [10].

Though the role of intestinal microflora in regulating immune response and maintaining host barriers against infection have long been recognized, the specific mechanism by which the gut microbiota may modulate GVHD is not well understood. Using a mouse model, Jenq et al. found a marked loss in microbial diversity in mice with GVHD. They noted expansion of certain bacterial subpopulations, especially *Lactobacillales*, in mice with GVHD [5]. An increased abundance of *Enterobacter* came at the expense of other commensals such as *Clostridia* and *Bacteroides* species. They observed that the expansion of the *Lactobacillales* was not the culprit for the development of GVHD since mice depleted of *Lactobacillales* developed GVHD that was more severe, pointing to a role of the *Lactobacillales* as a disease modulator. Re-introduction of gut commensals in these hosts reduced GVHD mortality. Subsequent analysis in human subjects identified similar dysbiosis in patients with gastrointestinal GVHD, with increase in *Lactobacillales* and decrease in *Clostridiales* associated with gut inflammation [18].

Interestingly, several studies have suggested an association between GVHD and *C. difficile* infection in allogeneic SCT recipients. Chakrabarti et al. demonstrated an association between CDI and severe GVHD [19]. Similar results were produced by Dubberke et al. who demonstrated a temporal relationship between CDI and GVHD [20]. Patients with CDI, including mild infection, had a higher likelihood of subsequently developing new-onset GVHD and new-onset gut GVHD. Trifilio et al. showed that SCT recipients who developed CDI were more likely to be complicated by severe GVHD at day 60 and day 100 after transplant [21]. The association between CDI and subsequent GVHD may be related to either the direct intestinal inflammation caused by *C. difficile*, or to the severe loss of intestinal microflora causing both processes.

Since diet affects the composition of the intestinal microbiome [22], it follows that certain diet contribute to the development of GVHD. Enteral feeding has been shown to protect against GVHD [23]. Mice and human subjects receiving total parenteral nutrition exhibited increases in IFNγ and reduction in IL-4, IL-10, gut CD4+ cells [24, 25], a pattern of immune responses that are pro-inflammatory.

Changes in the intestinal metabolism and microbiota-derived metabolites are likely the mechanisms underlying the role of the intestinal microbiota in regulating GVHD. Butyrate, a short chain fatty acid (SCFA) has been shown to the STAT3-dependent pathways of both the innate immune and allo-stimulatory functions of antigen presenting cells. These are accomplished through upregulating indoleamine-2,3-dioxygenase (IDO) [26, 27]. High levels of IDO mediate immune suppression through two mechanisms. First, they deplete tryptophan needed for the metabolism of donor T cells, resulting in apoptosis of these allo-reactive T cells. Second, they stimulate Treg generation [28, 29].

Modulation of the intestinal microbiota may also be exploited to treat acute GVHD. In a small pilot study, Kakihana et al. performed fecal microbiota transplant (FMT) from healthy donors on four patients with steroid-refractory intestinal GVHD [30]. All four patients responded, three achieved a complete remission and one partial remission with FMT and concurrent corticosteroids.

## Conclusions

There is a growing understanding of the profound ways in which intestinal microbiota interact symbiotically with the human host to influence health. Shifts in the microbiome are increasingly seen to influence morbidity and mortality in stem cell transplant recipients. The loss of bacterial diversity, expansion of certain species at the expense of other commensals, and changes in inflammatory regulation shape the incidence and severity of many post-transplant complications. A new paradigm is required in management of the microbiome of SCT recipients, one that is perhaps less reliant on broad-spectrum antimicrobials in favor of a more targeted approach to the patient’s specific enterotype.

Since intestinal dysbiosis occurs after SCT, the role of prebiotics and probiotics to preserve the diverse commensal microflora is being researched. Prebiotics are indigestible carbohydrates, such as plant oligo- and polysaccharides, that are broken down exclusively by the gut flora to produce SCFA [31, 32]. SCFAs are a major source of energy for colonocytes. Peng et al. showed that butyrate, a SCFA, plays an important role in maintaining the intestinal wall barrier; increasing trans-epithelial electrical resistance and inulin permeability [33]. In mouse models, SCFAs were shown to help regulate the size and function of the colonic regulatory T cells [32]. Probiotics, live micro-organisms that mimic commensal microflora, have been used to restore intestinal microbiota. Use of one probiotic agent, *Lactobacillus*, has been found to dramatically reduce *Enterococcal* growth in the post-SCT period [19]. These findings were supported by Gerbitz et al., who showed that administration of *Lactobacillus rhamnosus* improved survival post-SCT, reduced incidence of graft-versus-host disease, and decreased translocation of enteric bacteria [34]. Additionally, SCFAs promote the resolution of intestinal inflammation via the G protein-coupled receptor Gpr43, which in turn helps maintain the intestinal barrier [35, 36].

Replacement of commensal microflora to maintain a stable microbiologic landscape may also significantly reduce morbidity from GVHD. There is nascent data to support this practice: Jenq et al. showed that reintroduction of *Lactobacillus johnsonii* after marrow transplant reduced mortality from GVHD in mice [5]. Although this phenomenon needs to be studied further in humans, it is conceivable that early restoration of diverse bowel microflora may prevent GI complications. A prebiotic regimen, possibly individualized based on a patient’s baseline enterotype, could become an increasingly widespread practice in transplantation medicine. Dietary adjustments and probiotics may help maintain microbiologic homeostasis and preserve intestinal mucosa in the post-transplant period as well, working in synergy with prebiotics.

The gut microbiome can also be maintained through FMT, particularly after severe *Clostridium difficile* infection. Although autologous FMT is currently actively being investigated, it may theoretically be more efficacious using same-donor combined FMT and SCT. If a loss of microbial diversity and resultant mucosal inflammation leads to significant morbidity in SCT recipients, then restoration of the microflora by FMT may prove beneficial. The pathophysiology of GVHD, in particular, is mediated by donor T-lymphocytes and may be partly mitigated through same donor FMT since the donor T cells are more likely to be tolerant to the donor intestinal microbiota than the recipient microbiota.

Approaches to antibiotic treatment should also be re-examined. Broad-spectrum agents may paradoxically result in overgrowth of harmful genera and promote antibiotic resistance. More targeted agents may minimize infectious risk while preserving the bulk of the intestinal flora, whose presence helps maintain health. A patient’s individualized pre-transplant microbial landscape may be used to tailor antibiotic regimens, so as to minimize pathologic disruptions to gut flora.

Building on our emerging understanding of the microbiome to modify clinical practice will be a major challenge and opportunity in SCT medicine in the years to come. There is significant scope for clinical studies to examine the effects of changes in pre- and post-transplant management, through antibiotic adjustments, microbial repletion, and FMT.

## Abbreviations

GI: gastrointestinal
SCT: stem cell transplant
GVHD: graft-versus-host disease
MAMPs: microbial-associated molecular patterns
PRRS: pattern recognition receptors
IL: interleukin
CDI: *Clostridium difficile* infection
MRSA: Methicillin-resistant *Staphylococcus Aureus*
SCFA: short chain fatty acid
FMT: fetal microbiota transplant
